# Lancing the Gums in Dentition

**Published:** 1863-02

**Authors:** 


*Lancing the Gums in Dentition.*—From a review of Dr. Jacobi's recent work on Dentition, (Amer. Med. Monthly,) we extract the following judicious observations:

While we are on the topic of 'lancing the gums,' a word may not be out of place as to the *modus operandi* of the relief obtained by incisions. The growing tooth is enveloped by a saccular membrane, continuous with the periostem which surrounds the neck and fang, and secretes (afterward) the cementum. This dental periosteum is a highly important structure, well worthy of far more study than it has received. All judicious dentists recognize and respect it, for they know how highly vitalized it is, and how fruitful of mischief it becomes in disease. It is to this organ that are due those long-continuing and deep-seated facial neuralgias, which, like many more obscure symptoms of nervous derangement, are often suddenly and permanently relieved by extracting a diseased tooth. Now this membrane, so powerful in the adult, and so persistent in its bad effects, but so easily deprived of all harmful power, is the inclosure within which the growing tooth is formed ; and surely it is no stretch of credulity to believe that any fault or imperfection in this delicate process may as readily disorder the extremely sensitive nervous system of a child as may another form of diseased action in the same organ disturb the action of the adult system. The purpose, therefore, in cutting, is to relieve any undue tension of this membrane, and to do this, of course the knife must penetrate to the tooth. Prof. J.'s fancy about *scratching* the enamel is one of those points which show that his lectures are based more on theory than on observation. Frequently, as was the fact in the case above related, the relief is required and obtained by incising, some time before the tooth finally appears through the gum. The Professor may disdain the term, but the nurses are not so far out of the way—disturbance frequently occurs when the teeth are 'breeding.'

It will be very difficult to convince practical men, however it may be with mere theorists, that free incisions in the swollen gum do not frequently afford immediate relief, not only to local pain but also to more or less severe symptoms of a reflex character, having their starting point in the great local disturbance of nutrition of the dental nerves.

